# Automatic detection of n-degree family members

**DOI:** 10.3389/fgene.2025.1708315

**Published:** 2025-12-12

**Authors:** Emil M. Pedersen, Jette Steinbach, Carsten B. Pedersen, Andrew J. Schork, Morten D. Krebs, Bjarni J. Vilhjálmsson, Florian Privé

**Affiliations:** 1 National Centre for Register-Based Research, Aarhus University, Aarhus, Denmark; 2 Institute of Biological Psychiatry, Mental Health Center - Sct Hans, Copenhagen University Hospital, Copenhagen, Denmark; 3 Lundbeck Foundation Initiative for Integrative Psychiatric Research, iPSYCH, Aarhus, Denmark; 4 Bioinformatics Research Centre, Aarhus University, Aarhus, Denmark; 5 Novo Nordisk Foundation Center for Genomic Mechanisms of Disease, The Broad Institute of MIT and Harvard, Cambridge, MA, United States

**Keywords:** pedigree analysis, kinship matrix, graph theory, trio data, family-based studies, genetic epidemiology, R package

## Abstract

Summary: Family-based genetic studies often require the identification of relatives up to a specified degree, but existing tools are either restricted to second-degree relatives, return entire connected pedigrees, or require multiple pre- or post-processing steps. We implemented five new functions, namely, *prepare_graph, get_kinship, graph_to_trio, get_relations*, and *Relation_per_proband_plot,* in the R package LTFHPlus to address these limitations. *prepare_graph* constructs a directed graph from population-level trio data using the *igraph* package and supports attaching additional attributes to individuals. From this graph, relatives of arbitrary degree can be identified efficiently. *get_kinship* calculates a kinship matrix for all individuals in a (sub)graph, and *graph_to_trio* reconstructs trio information from identified families, enabling downstream use with other pedigree tools. In addition, familial relations can be labelled from the graph using the function *get_relations*, and the total and average of each relation per proband can be plotted using *Relation_per_proband_plot*. Using the publicly available minnbreast dataset, we constructed a graph containing 28,081 individuals and 30,720 familial edges. Across 1,000 repetitions, the median run-time for identifying all relatives up to the third degree for 500 randomly selected individuals was 0.03 s, and kinship matrix calculation had a median run-time of 1.57 s (single-threaded execution). These functions provide a reproducible, scalable, and interoperable solution for integrating family information into genetic analyses.

## Introduction

All fields interested in genetics have roots in studying resemblance among relatives, making efficient handling of large-scale pedigree data critical (Sham, 1996; Polubriaginof et al., 2018). Manually constructing family trees for individuals in large biobanks, population registers, or other study populations is tedious and error-prone. Flexible, well-documented computational tools for constructing, managing, and querying genealogical data that are integrated within analytical packages are needed to connect classical pedigree methods with modern molecular genetics. Only limited resources have recently been dedicated to the analysis of family data and pedigrees in combination with currently popular genotype data.

**FIGURE 1:**
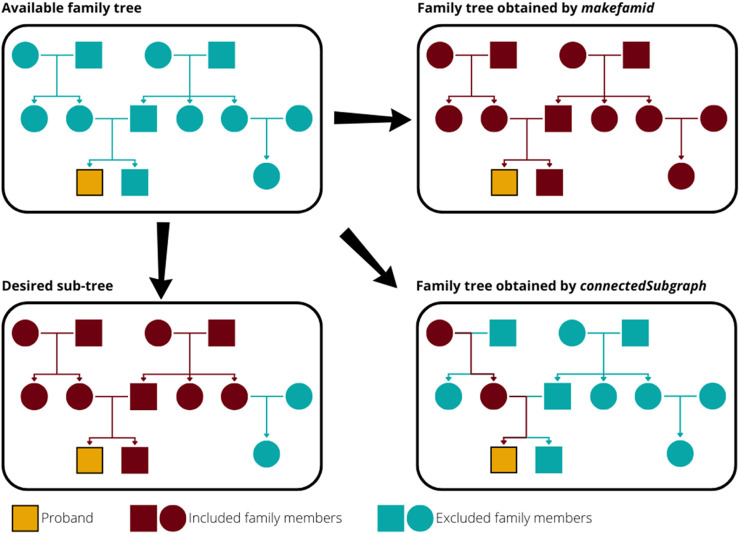
Comparison of family members identified by different R functions. The desired output is all relatives up to the second degree of the highlighted proband. The function *makefamid* from kinship2 returns all connected individuals, regardless of the degree of relatedness. The function *connectedSubgraph* from FamAgg returns only the individuals on the shortest path connecting two specified individuals (here, the proband and the maternal grandmother), thereby omitting other relatives not required to connect them. This example is equivalent to the maternal lineage identified by the function *gen.lineages* from GENLIB.

Existing tools have important limitations. Functions for constructing proband-specific family trees from trio data (proband, mother, and father identifiers) are typically embedded within specialised packages, restricting interoperability. For example, the FamAgg (Rainer et al., 2016) package offers *connectedSubgraph*, which identifies the smallest possible subgraph connecting two individuals, but ignores any relatives not directly needed for connectivity (see Figure 1). The Pedixplorer (Le Nézet et al., 2025) package supersedes Kinship2 (Sinnwell et al., 2014) and includes functions such as *makefamid* to generate a family identifier (see Figure 1). However, it returns the largest possible family tree and is not intended to find proband-specific subtrees. The closest method is the function *useful_inds* from Pedixplorer, but the package focuses on the visualisation of pedigrees and lacks analysis functionality. Similarly, GENLIB (Gauvin et al., 2015) provides utilities for identifying relatives and filtering by degree, but its proband definition (no children) and lack of descendant identification require *ad hoc* censoring of the data. Pedtools (Vigeland, 2021) can identify relatives up to second degree, but each role relative to the proband has its own function. Other tools, such as PERSEUS (Pradas et al., 2024), are online platforms, which makes them incompatible with analysis of human data in a secure compute environment. Although these tools could identify relatives up to the second degree, they require integrating some preprocessing or chaining of functions.

A single function for extracting *n*th-degree relatives from recorded trio data remains important, especially if it can interoperate with family scoring algorithms for GWAS applications or prediction. In this study, we implemented the five new functions, namely, *prepare_graph*, *get_kinship*, *graph_to_trio, get_relations*, and *Relation_per_proband_plot*, in the existing R package LTFHPlus (GitHub - EmilMiP/LTFHPlus, 2025) that uses efficient path counting algorithms developed by the package igraph (igraph, 2023) to automatically identify family members and label them in typical familial relations. The first function, *prepare_graph*, constructs a (directed) graph from population-level trio input and can attach additional information to each individual. Using functions from the R package igraph (igraph, 2023), manipulations can then be performed on this graph to create neighbourhood graphs, i.e., to identify all family members of degree n and closer efficiently. Identification of n-degree relatives is implemented in the wrapper function *get_family_graphs*. The second function, *get_kinship*, constructs a kinship matrix from a (sub)graph. The third function, *graph_to_trio*, recovers the trio information used to generate a graph, which makes it possible to utilise properties of existing packages on the identified families, such as pedigree plotting with Pedixplorer. Finally, a function called *get_relations* has been added to label relatives into typical relationships, such as full and half relations, parents, great-great-grandparents, nieces/nephews, cousins, second cousins twice removed, and more. A full list of identifiable relations can be found in the documentation. The average or total of each relation per proband can be visualised using the function *Relation_per_proband_plot*, which allows for a summary visualisation of the identified families.

## Methods

All functions are implemented in R using the igraph (igraph, 2023) package, which allows for a very efficient construction and manipulation of graphs with millions of nodes and edges. We implement the function *prepare_graph* that constructs a directed graph from three variables: the personal ID for the target individual along with the personal IDs for the mother and father. The directed graph is obtained through the following steps:

1. Data formatting and quality control, i.e.,
  ○ Ensuring IDs are strings
  ○ Substituting missing values with NAs
  ○ Converting to the data format used by igraph
2. Identifying parental links
3. Adding edges between full siblings
4. Identifying and adding individuals with no relatives (i.e., isolated nodes)
5. Adding additional individual information (i.e., attaching attributes to nodes)

Once the full population graph is constructed, the function *make_neighbourhood_graph* (alias *make_ego_graph*) from the igraph package can be used to identify all family members of degree n. Given the proband ID (name of a node) or a list of proband IDs, a single n-degree neighbourhood graph is created for each proband. Each neighbourhood graph is centred around the proband, and a proband can also be included as an n-degree relative in the neighbourhood graph for a different proband. A wrapper function that formats the resulting neighbourhood graphs is provided in *get_family_graphs*. Once the desired sub-graph(s) have been obtained, additional manipulations can be performed in order to add, extract, or modify attributes for each identified node in the neighbourhood graph (which corresponds to identifying family members) or to add or remove nodes and edges (which corresponds to adding or removing family members or their relations from the (sub) graph).

In addition, we introduce a second new function in LTFHPlus, *get_kinship*, which calculates the kinship matrix for all individuals present in a (neighbourhood) graph. The kinship matrix is based on the distance between nodes (individuals) in the graph, and it is calculated as $0.5^{d\left(i,j\right)}\times C$ , where $d\left(i,j\right)$ is the shortest distance between individual i and j through a most recent common ancestor node and C is a constant. Hence, it is a kinship-by-path-counting estimator. The variable C defaults to 1 but may represent variables such as the heritability of a phenotype.

Next, we also introduce a function called *graph_to_trio*, which takes a graph and reconstructs the trio information used to create it. This step requires sex as an attribute in the graph; however, it allows for the identification of families of arbitrary degrees and for converting the identified families back into a format that is usable by existing pedigree packages. In the trio format, the identified families can be used by existing pedigree packages, such as pedigree plotting with Pedixplorer.

**FIGURE 2:**
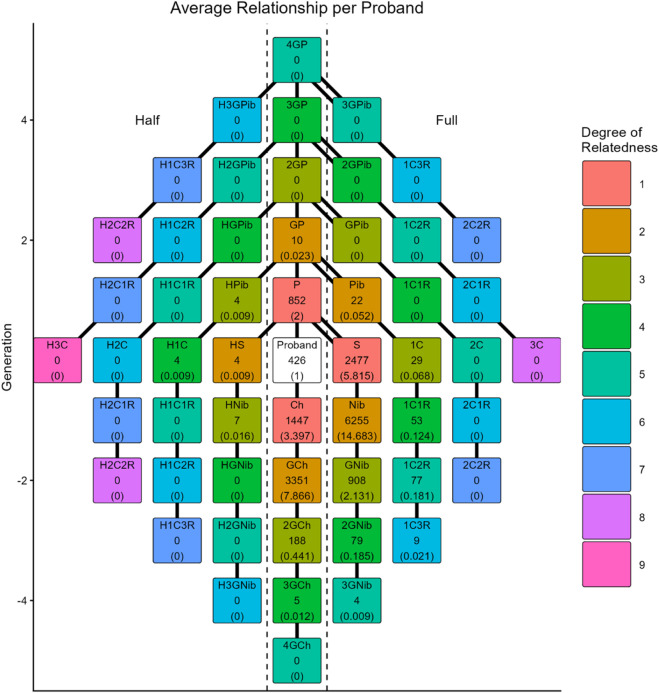
Visualisation of the total and average number of family relations per proband in the minnbreast data. The labels in a square represent the relative’s label, e.g., Ch for child and 1C2R for first cousin twice removed, followed by the total number observed and, finally, the average number per proband in parentheses. As an example, “Pib” is short for “pibling,” i.e., parent’s sibling (aunts/uncles), 22 are identified, which is an average of 0.052 per proband.

Finally, we introduce a function called *get_relations*, which calculates the number of generations up and down between every member of a family graph and labels them with commonly used family labels. Combined with the *Relation_per_proband_plot*, an overview of the identified family relations can be visualised. See Figure 2 for an example of the 426 probands from the minnbreast data. For Figure 2, we identified all relatives up to the 10th degree, labelled all identified relatives, and plotted the total number and average number of identified relations. The labelling favours horizontal family members identifiable from the great-great-great-great-grandparents of a proband and down the family tree, which means that the relations labelled are not exhaustive past the fifth degree and include no relations past the ninth degree.

## Results

In the publicly available minnbreast trio data (Sellers et al., 1995) distributed using the Kinship2 package (Sinnwell et al., 2014), we constructed a graph with all available data using *prepare_graph*, resulting in 28,081 nodes (individuals) and 30,720 edges (familial relations). We then randomly selected 500 individuals, extracted all family members up to the third degree, and applied *get_kinship* to each identified family. We repeated this process 1,000 times and recorded the run-times with *system.time* (1/100th second precision). The median run-time of identification of relatives up to the third degree was 0.03 s, and the median run-time of *get_kinship* was 1.57 s. First and third quartiles are provided in Table 1. If it is necessary to perform these calculations on millions of probands, the calculations can be carried out in parallel using a split-apply-combine strategy, with chunk sizes chosen such that each chunk will fit within the available resources.

**TABLE 1:** Median run-times (seconds) for the extraction of relatives up to the third degree and kinship matrix calculation (1,000 repetitions).

Task	First quartile (s)	Median run-time (s)	Third quartile (s)
Extraction of relatives (≤3rd degree)	0.02	0.03	0.03
Kinship matrix	1.44	1.57	1.73

Pedixplorer offers comparable functionality using the *useful_inds* function. We measured the run-time, cumulative RAM allocation, and peak RAM usage for the identification of all third-degree relatives of the proband in the first family (famid of 4) in the minnbreast data using the *useful_inds* function. The run-time of a single family using *useful_inds* was 68.2 s (compared to a median run-time of just 0.03 s with igraph-based extraction of 500 families of the same size). Using the R package peakRAM (GitHub - tpq/peakRAM, 2025), the reported peak RAM usage for the identification of all family members up to the third degree for a single individual was 36,097.4 MiB with *useful_inds*, and the system reported a peak memory allocation of 99%. Notably, R does not measure *currently allocated memory*, which means methods that measure RAM usage in R instead track each allocation request or the cumulative requested memory. peakRAM falls into the latter category, indicating that it can exceed the RAM available to the system.

peakRAM showed peak RAM allocation of 1.5 MiB on a chunk of 500 families using the igraph-based approach employed by LTFHPlus.

All analyses were conducted on a Lenovo ThinkPad laptop with an AMD Ryzen 7 PRO 7840U processor (1 core used, no parallelisation), 32 GB of available system RAM, running Windows 11 with R version 4.4.2, LTFHPlus v2.2.0, and igraph v2.1.4.

The minnbreast data record the proband of each family. Using *get_family_graphs*, we identify all family members up to the 10th degree for each proband. Then, we label all identified family members with a relationship label using *get_relations* and finally plot the total and average number of each identified relationship label for all probands in the minnbreast data. This summary information is visualised using the function *Relation_per_proband_plot* and is provided in Figure 2.

## Discussion

The automatic identification of family members presented in this study has three benefits: it removes the potential for errors when identifying family members, preserves individual-level family member information, and enables the efficient construction of kinship matrices. The function *prepare_graph* can store information for each individual, which is preserved during the construction of family trees of a suitable degree. Preserving information is helpful when the family trees are used to obtain family-based variables, such as binary family history indicators or family liabilities (Krebs et al., 2023; Pedersen et al., 2022). It also allows for the storage of other parameters on an individual family member level.

The second advantage of the approach is the efficient construction of kinship matrices for all identified families. Kinship matrices are applied in many settings; for example, they can be used to account for degrees of relatedness in mixed models (Rabe-Hesketh et al., 2008) and construct covariance matrices for liability threshold models conditional on family history (Pedersen et al., 2022). Hence, *get_kinship* can increase efficiency in a broad spectrum of applications by removing the need to convert to other input formats.

A third contribution of the work is the ability to label relatives into standard familial relationships using the *get_relations* function and visualise a summary of the relations using *Relation_per_proband_plot*. These additions assist in better understanding the identified families and facilitate a clear visual summary of them. Such visual summaries are useful when analysing population-level registers or large biobanks, where family structures may be complex and vary substantially across participants or where different categories of participants are being compared.

A comparison with Pedixplorer, which offers a similar functionality in *useful_inds*, showed the advantage of the igraph-based implementation utilised by LTFHPlus. In terms of run-time and RAM footprint, the graph-based approach performed better and demonstrated population-level scalability.

A particularly important application is the estimation of family (genetic) liabilities, which is the main purpose of the LTFHPlus package (Pedersen et al., 2022; Pedersen et al., 2023). Until now, the identification and construction of family trees and kinship matrices were major hurdles. With the automatic identification of family members and the preservation of individual family member information, we have now removed this barrier, minimised the potential for errors, and reduced computation time.

The kinship calculation has some limitations. First, the zygosity of twins is not considered (i.e., monozygotic twins are identified as simple siblings). Second, the shortest path through the most recent common ancestor is not inbreed-aware (as for consanguineous families); however, the shortest path estimate is comparable to inbreeding-aware estimates in small families. Alternatively, it is possible to convert the identified family members up to degree n back into trio data and use a different kinship calculator.

Together, the presented functions enable the automatic, accurate, and efficient extraction of relatives of arbitrary degree; the construction of kinship matrices; the labelling and visualisation of summaries of the identified family members; and the integration of family information into downstream analyses. We expect these tools to greatly facilitate the adoption of family-based approaches in large-scale genetic studies.

## Data Availability

The original contributions presented in the study are included in the article/supplementary material; further inquiries can be directed to the corresponding author.
